# Volumetric thermo-convective casson fluid flow over a nonlinear inclined extended surface

**DOI:** 10.1038/s41598-023-33259-z

**Published:** 2023-04-18

**Authors:** Muhammad Shuaib, Muhammad Anas, Hijab ur Rehman, Arshad Khan, Ilyas Khan, Sayed M. Eldin

**Affiliations:** 1 City University of Science and Information Technology, Peshawar, K.P.K, Pakistan; 2 Institute of Computer Sciences and Information Technology, The University of Agriculture, Peshawar, Pakistan; 3 Department of Mathematics, College of Science Al-Zulfi, Majmaah University, Al-Majmaah, 11952 Saudi Arabia; 4 Center of Research, Faculty of Engineering, Future University in Egypt, New Cairo, 11835 Egypt

**Keywords:** Chemistry, Energy science and technology, Engineering, Mathematics and computing, Physics

## Abstract

The thermophysical features of Casson fluid flow caused by a nonlinear permeable stretchable surface are assessed in the present study. The computational model of Casson fluid is used to define viscoelasticity, which is quantified rheologically in the momentum equation. Exothermic chemical reactions, heat absorption/generation, magnetic field and nonlinear volumetric thermal/mass expansion over the stretched surface are also considered. The proposed model equations are lessened by the similarity transformation to the dimensionless system of ODEs. The obtained set of differential equations are numerically computed through parametric continuation approach. The results are displayed and discussed via figures and tables. The outcomes of the proposed problem are compared to the existing literature and bvp4c package for the validity and accuracy purposes. It has been perceived that the energy and mass transition rate of Casson fluid increased with the flourishing trend of heat source parameter and chemical reaction respectively. Casson fluid velocity can be elevated by the rising effect of thermal, mass Grashof number and nonlinear thermal convection.

## Introduction

During last few years the importance of non-Newtonian fluids have been increased due to its significant application in the field of engineering, aerodynamic and paperwork, manufacturing, coating, polymer processing, and so on. Mud, blood, paint, polymer solutions are some of the materials that display this property. Due to the complexity of non-Newtonian fluids in physical nature there is no individual model that can accurately represent all of its characteristics. The non-Newtonian fluids have elastic solid like properties and Casson fluid is one of the example of such fluids. Gbadeyan et al.^1^ modeled the Casson fluid with the effect of variable thermal conductivity and viscosity which causes a shear thinning effect in the fluid. Akbar & Khan^2^ demonstrated that the effect of concentration and thermal is due to both pressure and temperature gradient in porous medium. Xu et al.^3^ used the parametric continuation approach to analyze an incompressible steady power law NF containing gyrotactic microbes flowing between parallel plates with energy conversion. Connective tissue that covers the outside wall of the micro vessel transfers heat was introduced by Shaw et al.^4^ at the surface followed by the convection of heat during atherosclerosis, hyperthermia, and other illnesses, in which diffusion and heat flux are critical. Adeosun et al.^5^ exposed the constant flow of a reactive fluid through a saturated porous material and observed that the nonlinear convective parameter enhanced both velocity and temperature profiles.

The magnetohydrodynamics (MHD) fluid flow has so many applications in disciplines like pharmacology, jets, and chemical industries. Due this wide range of applications the researchers diverted their attention towards MHD affected flows. The analysis of MHD Casson fluid is explored by Abo-Dahab et al.^6^ through a porous medium over extended surface with suction/injection as well as the impact of chemical processes over a nonlinear surface. They concluded that the findings were consistent with actual outcomes. The effects of Casson fluid flow under the influence of MHD across a stretched surface is examined by Hayat et al.^7^. They derived the relevant model for the flow and found a series solution by using the homotopic approach. Sohail et al.^8^ contributed the behavior of thermal diffusion and examine that how can non-Newtonian fluid flow move over a nonlinear stretching surface. Ajayi et al.^9^ investigated the flow of non-Newtonian over horizontal, vertical, inclined and cone. In which energy is connected due to temperature of plastic dynamic viscosity. Mukhopadhyay et al.^10^ discovered the flow of non-Newtonian fluid on boundary layer and energy heat transmission over an extended permeable surface. It was noticed that by increasing the Casson parameter causes the decrease in velocity field and increase in temperature field. Alsaedi, et al.^11^ clarify how heat is transferred at the surface due to Casson fluid. Zaib et al.^12^ discussed the transfer of heat through permeable sheet under the viscous dissipation of Casson fluid in two dimension flow on boundary. Aneja et al.^13^ obtain the problems as Casson fluid used in square porous cavity. Mukhopadhyay^14^ presented heat transfer of non-Newtonian fluid over nonlinear stretched surface. Khan et al.^15^ observed viscous dissipation by neglecting the effects and examine mass transfer over stretching sheet using Casson fluid. Khan et al.^16^ investigate the effect of natural convection through moving plate having porous media due to buoyancy forces of temperature and concentration gradients.

Bukhari et al.^17^ observed the heat transfer of the fluid with in the magnetic and radiation effect which have established biochemical instruments for pulsation. Pramanik^18^ under the suction non newtonian fluid is observed over stretch surface. Ali et al.^19^ investigates non-Newtonian Casson fluid flow with pulsation and symmetrical constriction bumps on top and bottom walls. During the pulsation cycle, it is also seen that raising the value of the porosity parameter reduces wall shear stress. Ali et al.^20^ analysis the heat transfer in pulsing flow in a channel with several symmetric constrictions on the walls. Lorentz force and thermal radiation have an impact on the flow. The finite difference approach is used to solve the unstable governing equations, which have been simplified for low conductive fluids. Saqlain et al.^21^ disclosed the features of nonlinear free convective nanofluid flow of radiated Casson fluid along non-uniform heat sources or sinks. The thermophoretic effect and generalised Fourier’s and Fick’s laws to study heat and mass movement.

Mustafa et al.^22^ discusses heat and unsteady flow of Casson fluid over moving flat plate in parallel stream, using homotopy technique to find the solution of nonlinear partial differential equations in the entire spatial domain. Samrat et al.^23^ examined the limiting of Brownian moment and thermophoresis of magnetohydrodynamic-free convection flow along the higher section of the paraboloid of revolution and using suitable transformations, the governing equations that lead to this model boundary restrictions are compressed into ODEs.

Chemical reaction plays an important role in engineering, industries and biological sciences. There are two types of chemical reactions exothermic and endothermic. In exothermic process of reactions energy is released whilst in endothermic process the energy is observed from the environment. Keeping in view these applications of the chemical reactions Ganesh and Sridhar^24^ discussed the effect of chemical reaction towards MHD marginal layer. They found that the increasing values of the chemical reaction decreases the concentration profile. Dharmaiah et al.^25^ also concluded the same results, by investigating tha Hall and ion slip impact on magnet-litanium alloy nanoliquids. MHD radiative Casson-Nanofluid stream with chemical reaction through Darcy–Forchiemer medium is examined by Ganesh and Sridhar^26^. Sridhar et al.^27^ explore the study of MHD Williason nanofluid across a permeable medium past an extended sheet. The numerical approach of heat and mass transfer of MHD Casson fluid with chemical reaction is presented by Ganesh and Sridhar^28–30^, they observed that the increasing value of chemical reaction parameter is responsible of the decay in heat transfer rate. The chemical reactions in which the rate of reaction is proportional to the mth order of the concentration reactant is the mth order chemical reaction^31–33^.

From the very beginning the natural source of solar energy is used for the benefit of the humanity in the form of getting warmth and light. The Earth planet received a livable amount of $4\times 10^{15}$ m/W energy from the sun, which is almost 200 times more than the normal used energy. With the passage of time the humanity recognized the importance of solar energy and developed various procedures to store and to convert the solar energy into thermal energy. Zhang et al.^34^ analyzed the heat transfer effect in the employment of melting heat transfer. They found that the addition of nanoparticles can generate more energy. Activation of energy, incorporated by chemical reaction, is responsible for more heat transfer^35^. Shaheen et al.^36^ discussed the effects of variable characteristics on dusty Casson nanofluid with chemical reaction and Arrhenius activation energy. They mention that Arrhenius function is decreasing for the rising values of activation energy. The exothermic chemical reaction and activation of energy has been discussed by Ramzan et al.^37^ and found that in an exothermic chemical reaction, the energy of the reactants higher than the end products. Ramzan et al.^38^ discussed hydrodynamics and heat transfer with convective boundary conditions. They realized that solar thermalsystem has low efficiency due to working fluid’s weak thermophysical properties.

The aim of the present article is to model the nonlinear thermo-convective Casson fluid flow over an inclined extended surface, which will leads to a better understanding of fluid flow over nonlinear surfaces. The numerical findings of this study may be used for biological systems, textile engineering, pharmaceutical and polymers production industries. The physical importance quantities such as wall shear stress, mass and heat transfer rates are sketched through graphs and tables. All these quantities plays a vital role in manufacturing and engineering units.

Ramesh et al.^45^ investigated time-dependent injection/suction and slide effects in Casson-micropolar nanofluid flow. In the presence of chemically reactive activation energy, Madhukesh et al.^46^, investigated the bio-Marangoni convection flow of Casson nanoliquid across a porous medium. Using a modified Buongiorno’s model, Puneeth et al.^47^ investigated the three-dimensional mixed convection flow of hybrid Casson nanofluid via a non-linear stretching surface. Thammanna et al.^48^ looked studied couple stress Casson fluid flow in three dimensions past an unstable stretching surface with a chemical reaction. Some other similar studies are given in [?]. After reviewing the above mentioned literature, it found that no attention has been given to the effects of temperature and concentration on Casson’s fluid velocity. To over come this gap, the present research article is concerned with the modeling of Casson fluid flow over the surface of extended porous sheet with linear and nonlinear volumetric thermo-convection effects. The Navier-Stokes equations are coupled with temperature and concentration equations, by introducing linear and nonlinear thermo-convection terms in the momentum equation. The basic governing equations are transformed into a system of ODEs by using suitable transformations. The numerical results of the transformed system of ODEs are obtained by using two different numerical schemes, Parametric Continuation Method (PCM) and bvp4c package performed by using Matlab software. Both the results are figured out and found that they are in a very good agreement with each other. For further validation of the numerical schemes the obtained findings are tabulated and compared with previously published work, which gives an accurate result upto 3 decimal places. For sake of convergence, efficiency and accuracy the CPU time is also tabulated for both PCM and bvp4c.

## Mathematical formulation of the problem

**Figure 1:**
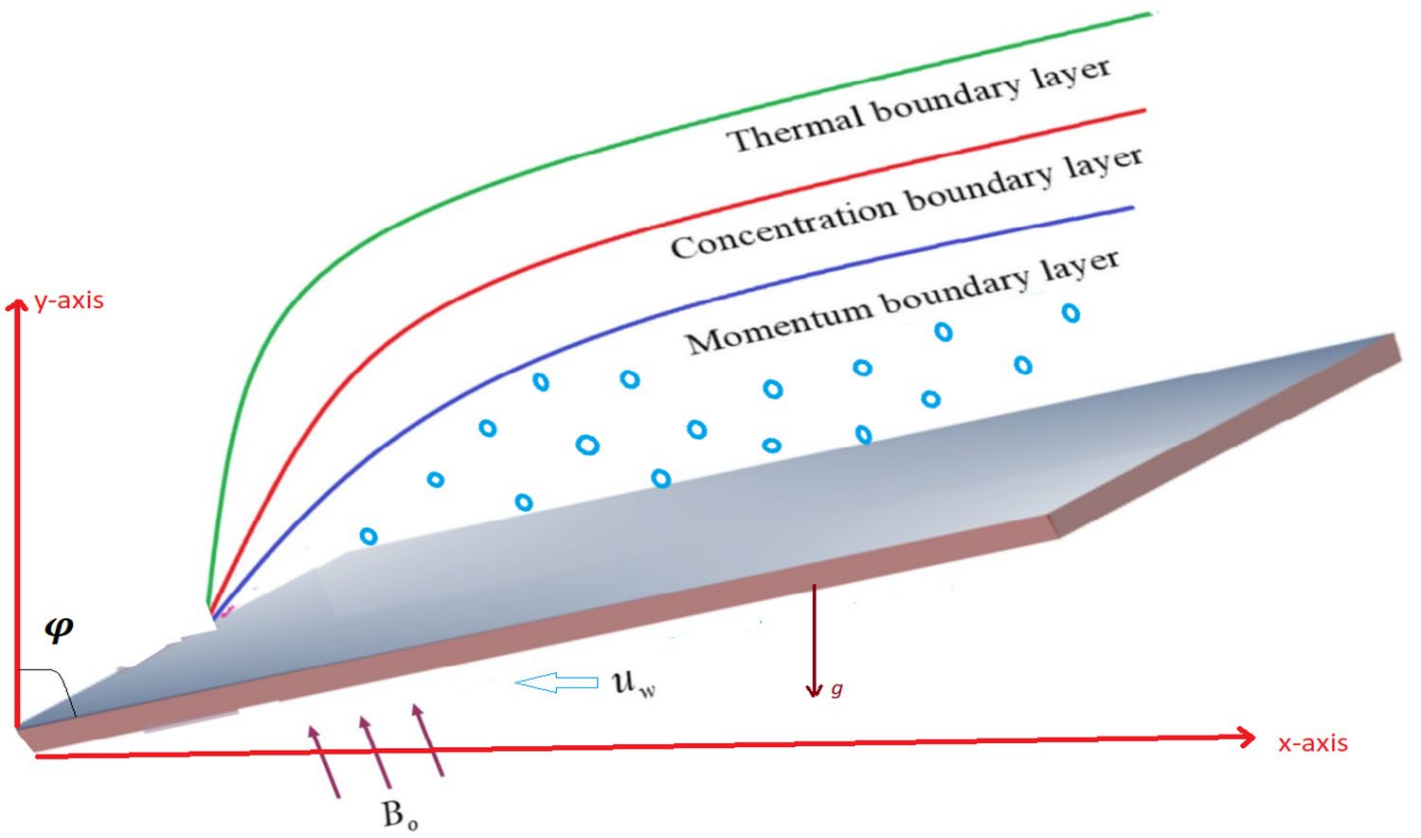
Geometry of the problem.

A linear and nonlinear thermo-convective Casson fluid will be considered in the region of $(y>0)$ over the surface of a nonlinear porous extended sheet with power-law given as $u_{w}(x)=bx^{n}$ and varying wall temperature $T_{w}=T_{\infty }+\delta x^{n}$ where $\delta$ is a positive constant. A change in magnetic field of strength $B(x)=B_ox^\frac{m-1}{2}$ and used in vertical direction. The induced electric and magnetic fields are ignored because of low magnetic Reynolds number. Coordinate system and physical sketch are shown in Fig. 1^6^.

1 $$\begin{aligned}{}&\frac{\partial u}{\partial x}+\frac{\partial v}{\partial y}= 0\text {,} \end{aligned}$$

2 $$\begin{aligned}{}&\left. \begin{array}{ll} &{}u\frac{\partial u}{\partial x}+v\frac{\partial v}{\partial y}=v(1+\frac{1}{\beta })(\frac{\partial ^{2}u}{\partial y^{2}})-\frac{v}{k}(1+\frac{1}{\beta })u-\frac{\sigma B^{2}(x)u}{\rho }\\ &{}\quad {+g(\beta _{T_{w}}(T-T_{\infty })+g\beta ^{2}_{T_1}(T-T_{\infty })^{2}+g\beta _{C_{w}}(C-C_{\infty })}\\ &{}\quad {+g\beta ^{2}_{C_1}(C-C_{\infty })^{2})\cos \varphi \text {,}}\\ \end{array}\right\} \end{aligned}$$

3 $$\begin{aligned}{}&u\frac{{\partial T}}{{\partial x}}+v\frac{{\partial T}}{{\partial y}}=\alpha \Bigg (\frac{\partial ^{2}T}{\partial y^{2}}\Bigg )+\frac{Q_{0}}{\rho c_{p}}(T-T_{\infty })+\tau \Bigg [D_{B}\Bigg (\frac{\partial T}{\partial y}\frac{\partial C}{\partial y}\Bigg )+\frac{D_{T}}{T_{\infty }}\Bigg (\frac{\partial T}{\partial y}\Bigg )^{2}\Bigg ]\nonumber \\&+\frac{v}{c_{p}}\Bigg (1+\frac{1}{\beta }\Bigg )\Bigg (\frac{\partial u}{\partial y}\Bigg )^{2}\text {,} \end{aligned}$$

4 $$\begin{aligned}{}&u\frac{\partial C}{\partial x}+v\frac{\partial C}{\partial y}=D_{B}\Bigg (\frac{\partial ^{2} C}{\partial y^{2}}\Bigg )+\frac{D_{T}}{T_{\infty }}\Bigg (\frac{\partial ^{2} T}{\partial y^{2}}\Bigg )-R_{0}(C-C_{\infty })\text {,} \end{aligned}$$

In the recent problem boundary conditions are as follows^6^:

5 $$\begin{aligned} \left. \begin{array}{ll} u=&{}u_{w}(x)=bx^{n},v=v_{w},T=T_{w}(x)=T_{\infty }+ax^{n}\text {,}\\ &{}D_{B}(\frac{\partial C}{\partial y})+\frac{D_{T}}{T_{\infty }}(\frac{\partial T}{\partial y})=0 \,\,{\text {at}}\,\, y=0\text {,}\\ &{}u\longrightarrow 0,T\longrightarrow T_{\infty },C\longrightarrow C_{\infty }\,\,{\text {as}}\,\,\longrightarrow \infty \text {,} \end{array}\right\} \end{aligned}$$

Non-dimensional transformations are as follows:

6 $$\begin{aligned} \left. \begin{array}{ll} &{}u=ax^{n}f^{'}(\eta )\text {,}\,\,v=-ax^{\frac{n-1}{2}}\sqrt{\frac{v}{a}}(\frac{n+1}{2}{f(\eta )}+\frac{n-1}{2}\eta f^{'}(\eta ))\text {,}\\ &{}\eta =\sqrt{\frac{a}{v}}x^{\frac{n-1}{2}}y\text {,}\,\,\theta (\eta )=\frac{T-T_\infty }{T_{w}-T_\infty }\text {,}\,\, \phi (\eta )=\frac{C-C_{\infty }}{C_{w}-C_{\infty }} \end{array}\right\} \end{aligned}$$

Using the system of non-dimensional transformations forms non-dimensional equations as follows:

7 $$\begin{aligned} \left. \begin{array}{ll} &{}(1+\frac{1}{\beta })f^{'''}-nf^{'2}+{\frac{n+1}{2}ff^{''}}{-(M+K)f^{'}}+Gr(\theta +\sigma _{1}\theta ^{2}){\cos \varphi }+Gc(\phi +\sigma _{2}\phi ^{2}){\cos \varphi }=0\text {,}\\ &{}\frac{1}{Pr}{\theta ^{''}}+\frac{n+1}{2}f\theta ^{'}-nf^{'}\theta +{\lambda \theta }+{Nb\theta ^{'}\phi ^{'}+Nt\theta ^{'2}}+(1+{\frac{1}{\beta }})Ecf^{''2}=0\text {,}\\ &{}\phi ^{''}+{\frac{n+1}{2}}Scf\phi ^{'}+{\frac{Nb}{Nt}}\theta ^{''}-RSc\phi =0\text {.}\\ \end{array}\right\} \end{aligned}$$

Boundary conditions will be:

8 $$\begin{aligned} \left. \begin{array}{ll} &{}f(0)=f_{w},f'(0)=1,\theta (0)=1,Nb{\phi '(0)+Nt\theta '(0)=0}, \,\,at\,\, \eta =0\\ &{}f'(\infty )\longrightarrow 0, \theta (\infty )\longrightarrow 0,\phi (\infty )\longrightarrow 0\,\, as\,\, \eta \longrightarrow \infty \end{array}\right\} \end{aligned}$$

Parameters used are given below:

9 $$\begin{aligned} \left. \begin{array}{ll} &{}Pr=\frac{\nu }{\alpha }\text {,}\,\, Sc=\frac{\nu }{D_{B}}\text {,}\,\,M=\frac{\sigma B_{0}^{2}}{a\rho c_{p}}\text {,}\,\,\lambda =\frac{Q_{0}}{u_{w}\rho c_{p}}\text {,}\,\,K=\frac{\nu }{ak_{0}}\text {,}\\ &{}Nb=\frac{D_{B}(C_{w}-C_{\infty })}{\nu }\text {,}\,\, Ec=\frac{u_{w}^{2}}{(T_{w}-T_{\infty })c_{p}}\text {,}\,\,R=\frac{R_{0}}{ax^{n-1}}\text {,}\\ &{} Nt=\frac{D_{T}(T_{w}-T_{\infty })}{T_{\infty }\nu }\text {,}\,\,f_{w}={\frac{-2v_{w}}{(n+1)a^{\frac{1}{n}}\sqrt{\nu u_{w}^{\frac{(2n-1)}{2n}}}}}\text {,}\\ &{}Gc=\frac{\rho gh^{2}\beta _{C0}(C-C_{\infty })}{(C-C_{\infty }){\mu UM}}\text {,}\,\,Gr=\frac{\rho \beta _{T_{\infty } gh^{2}RT_{\infty }^{2}}}{\mu UME}.\\ \end{array}\right\} \end{aligned}$$

The physical important parameters, such as mass transfer rate, heat transfer rate and rate of shear stress can be extracted by using the following definitions:

10 $$\begin{aligned} \left. \begin{array}{ll} Sh_{x}=\frac{xF_{m}}{D_{B}(C_{w}-C_{\infty })}\text {,}\,\,Nu_{x}=\frac{xF_{H}}{\alpha (T_{w}-T_{\infty })}\,\,\text {and}\,\, C_{x}=\frac{\sigma _{w}}{\rho u_{w}^{2}}\text {,} \end{array}\right\} \end{aligned}$$

where $Sh_{x}$, $Nu_{x}$, $F_{m}$, $F_{H}$ are Sherwood number, Nusselt number, mass and heat flux, respectively, $\alpha$ is the thermal conductivity and $\sigma _{w}$ is the wall shear stress. These can be defined as:

11 $$\begin{aligned} \left. \begin{array}{ll} F_{m}&{}=-D_{B}(\frac{\partial C}{\partial y})_{\,\,at \,\,y=0}\text {,}\\ F_{H}&{}=-\alpha (\frac{\partial T}{\partial y})_{\,\,at\,\, y=0}\text {,}\\ \sigma _{w}&{}=\mu _{B}(1+\frac{1}{\beta })(\frac{\partial u}{\partial y})_{\,\,at\,\, y=0}\text {.} \end{array}\right\} \end{aligned}$$

Incorporating Eqs. (6) and (11) into Eq. (10) to get

12 $$\begin{aligned} \left. \begin{array}{ll} Sh_{x}(Re_{x})^{-1/2}=-\phi '(0)\text {,}\,\,Nu_{x}(Re_{x})^{-1/2}=-\theta '(0)\text {,}\,\,Cf_{x}(Re_{x})^{1/2}=(1+\frac{1}{\beta })f''(0)\text {,} \end{array}\right\} \end{aligned}$$

where $Re_{x}=\frac{u_{w}x}{\nu }$ is the Reynolds number.

## Numerical Procedure by parametric continuation method

The fundamental steps involved in the solution of system of ODEs Eqs. (7) and (8) are as follow^39^:

**Step 1: Reduction of the system of BVP to the first order ODE:**

13 $$\begin{aligned} \begin{aligned} \left. \begin{array}{ll} F_{1}(\eta )&{}=f\text {,} \,\,F_{2}(\eta )=f'\text {,}\,\,F_{3}(\eta )=f''\text {,}\\ F_{4}(\eta )&{}=\theta \text {,}\,\, F_{5}(\eta )=\theta '\text {,}\\ F_{6}(\eta )&{}=\phi \text {,} \,\,F_{7}(\eta )=\phi '. \end{array}\right\} \end{aligned} \end{aligned}$$

By using Eq. (13) into the system of ODEs Eq. (7), one gets

14 $$\begin{aligned}{}&\left. \begin{array}{ll} &{}(1+\frac{1}{\beta })F'_{3}(\eta )-nF^{2}_{2}(\eta )+{\frac{n+1}{2}F_{1}(\eta )F_{3}(\eta )}-(M+K)F_{2}(\eta )\\ &{}\quad {+Gr(F_{4}(\eta )+\sigma _{1}F_{4}^{2}(\eta ))\cos \varphi +Gc(F_{6}(\eta )+\sigma _{2}F_{6}^{2}(\eta ))\cos \varphi =0\text {,}} \end{array}\right\} \end{aligned}$$

15 $$\begin{aligned}{}&\left. \begin{array}{ll} &{}\frac{1}{Pr}{F'_{5}(\eta )}+\frac{n+1}{2}F_{1}(\eta )F_{5}(\eta )-nF_{2}(\eta )F_{4}(\eta )+{\lambda F_{4}(\eta )}\\ &{}\quad {{+NbF_{5}(\eta )F_{7}(\eta )+NtF'^{2}_{5}(\eta )+(1+{\frac{1}{\beta }})EcF^{2}_{3}(\eta )=0\text {,}}} \end{array}\right\} \end{aligned}$$

16 $$\begin{aligned}{}&F'_{7}(\eta )+{\frac{n+1}{2}}ScF_{1}(\eta )F_{7}(\eta )+{\frac{Nb}{Nt}}F_{5}'(\eta )-RScF_{6}(\eta )=0\text {.} \end{aligned}$$

the corresponding boundary conditions are

17 $$\begin{aligned}{}&\left. \begin{array}{ll} &{}F_{1}(0)=f_{w},F_{2}(0)=1,F_{4}(0)=1,NbF_{7}(0)+N_{t}F_{5}(0)=0, \,\,at\,\, \eta =0\\ &{}F_{2}(\infty )\longrightarrow 0, F_{4}(\infty )\longrightarrow 0,F_{6}(\infty )\longrightarrow 0\,\, as \,\,\eta \longrightarrow \infty \end{array}\right\} \end{aligned}$$

**Step 2: Introducing the embedding parameter p:**

18 $$\begin{aligned}{}&\left. \begin{array}{ll} &{}(1+\frac{1}{\beta })F'_{3}(\eta )-nF^{2}_{2}(\eta )+{\frac{n+1}{2}F_{1}(\eta )(F_{3}(\eta )-1)p(\eta )}-(M+K)F_{2}(\eta )\\ &{}\quad {+Gr(F_{4}(\eta )+\sigma _{1}F_{4}^{2}(\eta ))\cos \varphi +Gc(F_{6}(\eta )+\sigma _{2}F_{6}^{2}(\eta ))\cos \varphi =0\text {,}} \end{array}\right\} \end{aligned}$$

19 $$\begin{aligned}{}&\left. \begin{array}{ll} &{}\frac{1}{Pr}{F'_{5}(\eta )}+\frac{n+1}{2}F_{1}(\eta )F_{5}(\eta )-nF_{2}(\eta )F_{4}(\eta )+{\lambda F_{4}(\eta )}\\ &{}\quad {{+Nb(F_{5}(\eta )-1)p(\eta )F_{7}(\eta )+NtF'^{2}_{5}(\eta )+(1+{\frac{1}{\beta }})EcF^{2}_{3}(\eta )=0\text {,}}} \end{array}\right\} \end{aligned}$$

20 $$\begin{aligned}{}&F'_{7}(\eta )+{\frac{n+1}{2}}ScF_{1}(\eta )(F_{7}(\eta )-1)p(\eta )+{\frac{Nb}{Nt}}F'_{5}(\eta )-RScF_{6}(\eta )=0\text {.} \end{aligned}$$

**Step 3: Differentiating by parameter p:**

21 $$\begin{aligned} V'=AV+R\text {,} \end{aligned}$$

where, A and R the coefficient and remainder matrices, respectively and

22 $$\begin{aligned} V=\frac{\partial F_{i}}{\partial \tau }\text {'} \end{aligned}$$

where $$i=1,2,\ldots 7.$$

**Step 4: Apply Cauchy problem and superposition principle**^40^:

23 $$\begin{aligned} V=aU+W\text {.} \end{aligned}$$

Solve the following two Cauchy problems for each component

24 $$\begin{aligned} U'&=AU\text {,} \end{aligned}$$

25 $$\begin{aligned} W'&=AW+R\text {,} \end{aligned}$$

putting the approximate solution Eq. (23) into the original Eq. (21), to obtain

26 $$\begin{aligned} (aU+W)'=A(aU+W)+R\text {,} \end{aligned}$$

**Step 5: Solving the Cauchy problems:** After applying forward difference approximations for Eqs. (24) and (25), to get

27 $$\begin{aligned} \frac{U^{i+1}-U^{i}}{\triangle \eta }&=AU^{i+1}\text {, or}(I-\triangle \eta A)U^{i+1}=U^{i}\text {,} \end{aligned}$$

28 $$\begin{aligned} \frac{W^{i+1}-W^{i}}{\triangle \eta }&=AW^{i+1}+R\text {, or}(I-\triangle \eta A)W^{i+1}=W^{i}+\triangle \eta R\text {,} \end{aligned}$$

The numerical solution of Eqs. (27) and (28) is possible only when the matrix $(I-\triangle \eta A)$ is nonsingular, that is

29 $$\begin{aligned} U^{i+1}&=(I-\triangle \eta A)^{-1}U^{i}\text {,} \end{aligned}$$

30 $$\begin{aligned} W^{i+1}&=(I-\triangle \eta A)^{-1}(W^{i}+\triangle \eta R)\text {.} \end{aligned}$$

Equations (29) and (30), gives the explicit iterative solutions for velocity, temperature and concentration fields.

## Results and discussions

**Figure 2:**
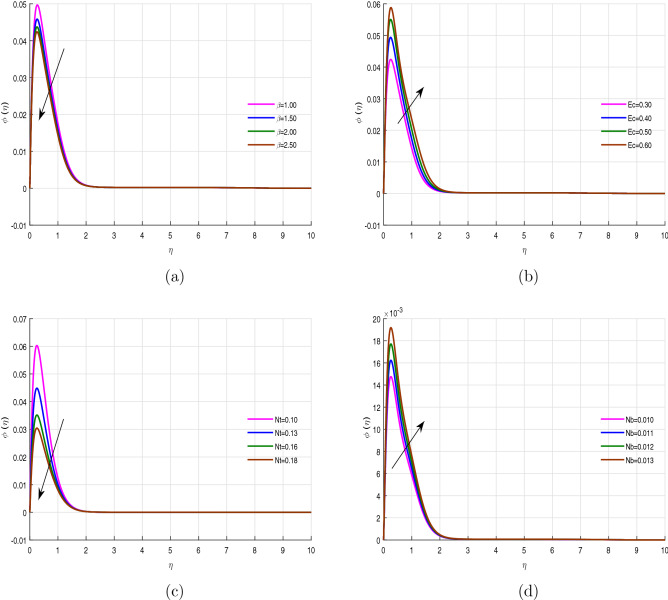
Concentration profile $\phi (\eta )$ for fixed values of physical parameters $\lambda =0.1$ $k=8.933$, $Pr=6.0$, $M=0.5$, $Gr=0.09$, $Gc=0.05$, $\sigma _1=1.1$, $\sigma _2=10.9$, $R=0.5$, $Ec=0.6$, $Nb=0.01$, $Nt=0.1$ $Sc=9.0$, $fw=1.0$, $\varphi =0.2$, $n=0.01$ and $\beta =2.5$.

**Figure 3:**
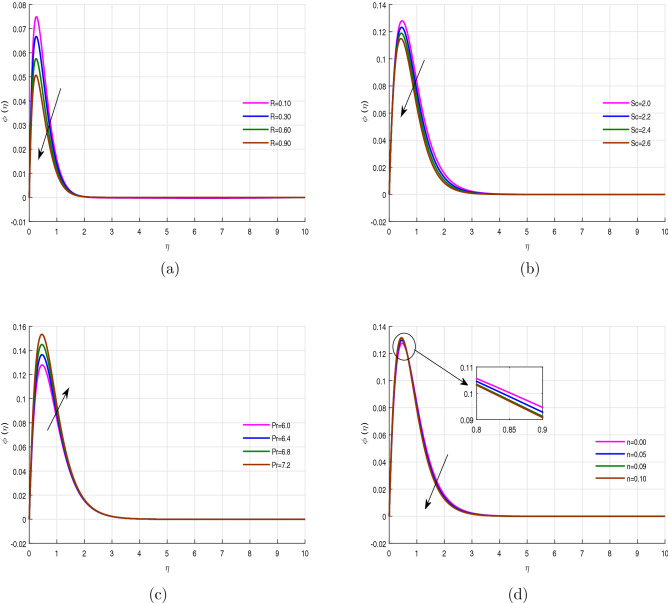
Concentration profile $\phi (\eta )$ for fixed values of physical parameters $\lambda =0.1$ $k=8.933$, $Pr=6.0$, $M=0.5$, $Gr=0.09$, $Gc=0.05$, $\sigma _1=1.1$, $\sigma _2=10.9$, $R=0.5$, $Ec=0.6$, $Nb=0.01$, $Nt=0.1$ $Sc=9.0$, $fw=1.0$, $\varphi =0.2$, $n=0.01$ and $\beta =2.5$.

**Figure 4:**
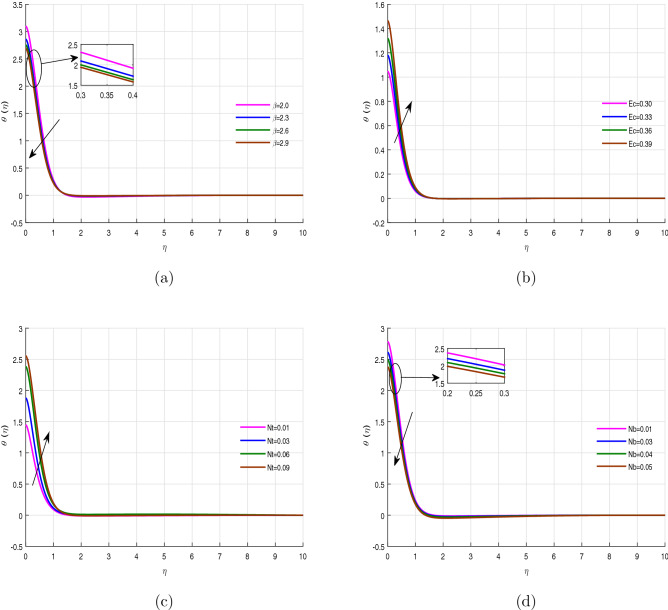
Temperature profile $\theta (\eta )$ for fixed values of physical parameters $\lambda =0.1$ $k=8.933$, $Pr=6.0$, $M=0.5$, $Gr=0.09$, $Gc=0.05$, $\sigma _1=1.1$, $\sigma _2=10.9$, $R=0.5$, $Ec=0.6$, $Nb=0.01$, $Nt=0.1$ $Sc=9.0$, $fw=1.0$, $\varphi =0.2$, $n=0.01$ and $\beta =2.5$.

**Figure 5:**
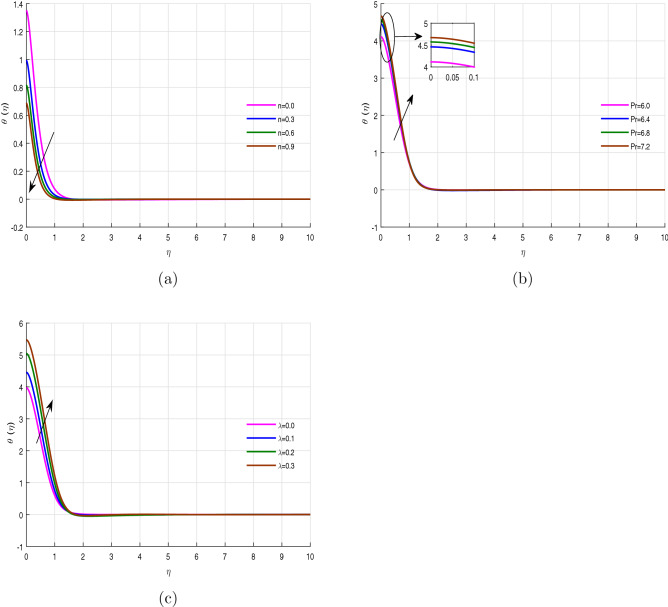
Temperature profile $\theta (\eta )$ for fixed values of physical parameters $\lambda =0.1$ $k=8.933$, $Pr=6.0$, $M=0.5$, $Gr=0.09$, $Gc=0.05$, $\sigma _1=1.1$, $\sigma _2=10.9$, $R=0.5$, $Ec=0.6$, $Nb=0.01$, $Nt=0.1$ $Sc=9.0$, $fw=1.0$, $\varphi =0.2$, $n=0.01$ and $\beta =2.5$.

**Figure 6:**
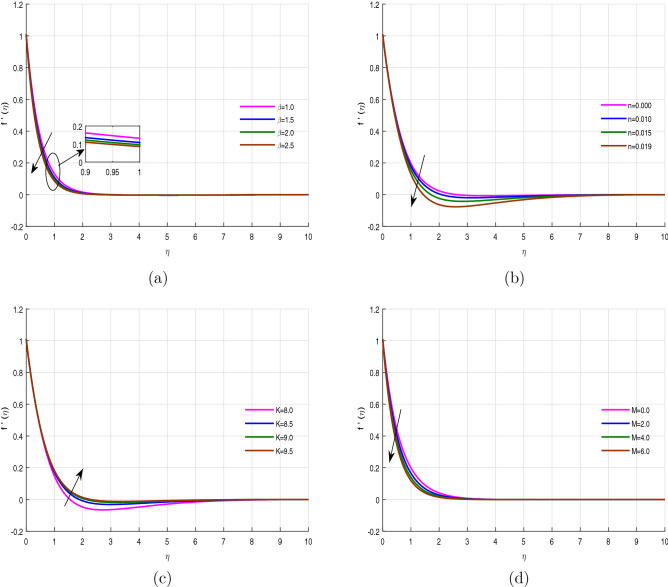
Velocity profile $f'(\eta )$ for fixed values of physical parameters $\lambda =0.1$ $k=8.933$, $Pr=6.0$, $M=0.5$, $Gr=0.09$, $Gc=0.05$, $\sigma _1=1.1$, $\sigma _2=10.9$, $R=0.5$, $Ec=0.6$, $Nb=0.01$, $Nt=0.1$ $Sc=9.0$, $fw=1.0$, $\varphi =0.2$, $n=0.01$ and $\beta =2.5$.

**Figure 7:**
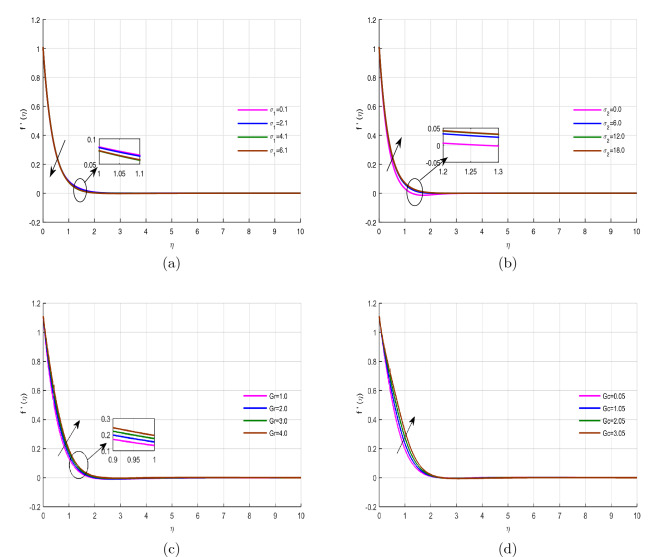
Velocity profile $f'(\eta )$ for fixed values of physical parameters $\lambda =0.1$ $k=8.933$, $Pr=6.0$, $M=0.5$, $Gr=0.09$, $Gc=0.05$, $\sigma _1=1.1$, $\sigma _2=10.9$, $R=0.5$, $Ec=0.6$, $Nb=0.01$, $Nt=0.1$ $Sc=9.0$, $fw=1.0$, $\varphi =0.2$, $n=0.01$ and $\beta =2.5$.

**Figure 8:**
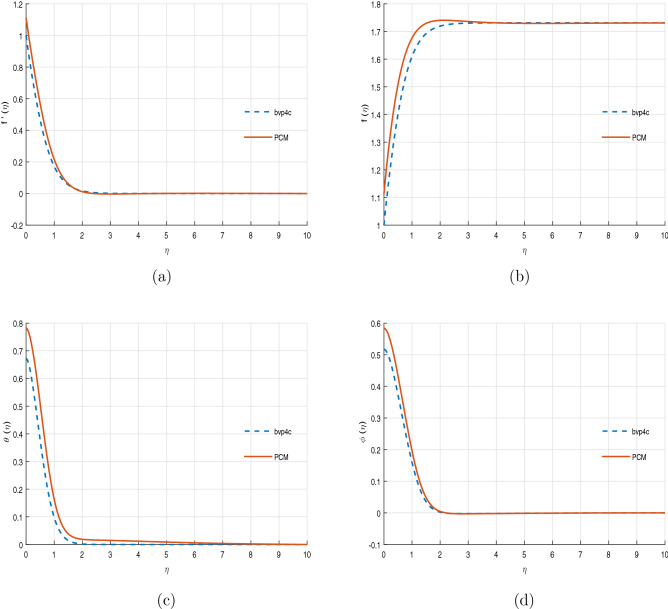
Comparative analysis of PCM and bvp4c methods.

The Casson fluid flow is modeled, in the form of Eq. (7) along with appropriate boundary conditions (8), over the surface of nonlinear volumetric thermo-convection extended porous sheet. The numerical investigation of the modeled equations are executed by using two different techniques PCM and *bvp*4*c*, and displayed by Figs. 2, 3, 4, 5, 6 and 7. The validity of both schemes is also presented graphically Fig. 8, which shows a contrast results for both numerical techniques. Further validation is carried out by comparing the numerical outcomes of PCM method with previously published work and tabulated the numerical values in Table 1. The physical interested quantities such as Sherwood number, Nusselt number and wall shear stress are illustrated through graphs (9–11) against different parameters.

The variation of concentration profile is depicted in Fig. 2a–d against different parameters such as Casson fluid parameter $\beta$, Eckert number *Ec*, thermophoresis parameter *Nt* and Brownian motion parameter *Nb*. The Casson fluid parameter $\beta$ has a dual behavior in experimental research, for $\beta =2$, it behaves like a non-Newtonian fluid, whilst for $\beta \longrightarrow \infty$, it becomes as a Newtonian fluid. Due to this dual nature of $\beta$, it is regarded as an important parameter both in experimental as well as in theoretical research. For the increasing values of $\beta$ range from 1.00 to 2.50, the fluid gradually takes the form of a non-Newtonian liquid and fluid will become more viscous, which results a decline in the concentration profile, as evident in Fig. 2a. Eckert number bear the ratio of kinetic energy to anthalpy (temperature difference). The impact of *Ec* on concentration profile $\phi (\eta )$ is displayed in Fig. 2b, and shows an increasing profile of $\phi (\eta )$ with the increasing values of *Ec*. This is due to the direct relation of Eckert number to the kinetic energy, which results much more transfer of fluid’s molecules from surface of the sheet. As shown earlier in “Mathematical formulation of the problem”, that thermophoresis parameter *Nt* and Brownian motion parameter *Nb* have a direct relation with temperature and concentration differences, respectively, which makes thermal boundary layer as smaller than concentration boundary layer as evident from Fig. 2c,d. And it can be reason out for a reduction in concentration profile $\phi (\eta )$ for the increasing values of *Nt* and having an enhancement in the profile for the increasing values of *Nb*.

Figure 3a–d exhibits the concentration profile for chemical reaction parameter *R*, Schmidt number *Sc*, Prandtl number *Pr* and nonlinear stretching parameter *n* for non-linear extended sheet. The chemical reaction *R* has the ability to exaggerate the inter molecules collision which results an increase in the internal heat generation of the system, due to which much more concentration is consumed. This consumption of concentration is reason out for a reduction in concentration profile for the increasing values of chemical reaction, see Fig. 3a. Figure 3b displays concentration profile behavior against Schmidt number. The mass transfer rate declines with the rising effect of Schmidt number, because the kinetic viscosity of fluid improves with the variation of *Sc*, which results in the declination of mass transition. The Prandtl number *Pr* represents the ratio of the viscous thickness to thermal diffusivity. The increase of Prandtl number leads to the reduction of concentration profile and concentration boundary layer, significantly, as evident from Fig. 3c. This is due to the increasing viscosity of the fluid, which brings about a reduction in the concentration profile. Figure 3d is provided to make a companion between the linear stretching sheet ($n=0$) and nonlinear stretching sheet ($n>0$). It is noted that the mass transference reduces with the action of nonlinear stretching parameter *n*. The linear stretching sheet shows maximum concentration compare to the nonlinear stretching sheet. The increase of the nonlinear stretching parameter makes the fluid’s particle moments parallel to each other and reduced the concentration profile with increased stretching forces, which results in increasing the pressure and strain motion.

Figure 4a–d revealed the performance of energy profile versus the variation of Casson fluid parameter $\beta$, Eckert number *Ec*, thermophoresis *Nt* and Brownian motion *Nb* respectively. The energy profile decline with the effect of Casson parameter, while boosts with the upshot of Eckert number *Ec* as shown in Fig. 4a,b. Because, the wall stretching enhances, while the specific heat capacity of fluid diminish with the variation of Eckert number, which causes the above scenario. Thermophoresis is the thermodynamics process developed due to the temperature difference within the fluid flow system, which takes away the hot fluid molecules into a cold region. Due to this reason the heat transfer rate is accelerated and can be depicted in Fig. 4c. Figure 4d highlights the energy propagation rate increases with the effect of Brownian motion *Nb*. The Brownian or random motion of the fluid particles causes the possibility of the collision among the particles, which releases the internal energy and gives a declination in temperature profile. Figure 5a–c displays the performance of the energy profile versus the variation of parameter *n*, Prandtl number *Pr*, and heat source parameter $\lambda$ respectively. In these figures, Fig. 5a shows a comparison of the linear stretching sheet ($n=0$) with the nonlinear stretching sheet ($n>0$). It can be observed that the temperature field decreases with the variation of parameter *n*, while enhances with the action of Prandtl number and heat source parameter $\lambda$. The thermal diffusivity of the fluid reduces with the influence of the Prandtl number, which is why the fluid temperature rises with its effect.

Figure 6a–d revealed the performance of the velocity profile versus the variation of Casson fluid parameter $\beta$, parameter *n*, porosity parameter *K*, and magnetic field *M* respectively. Figure 6a illustrated that the velocity profile declines with the effect of Casson parameter $\beta$, and *n*. Figure 6b provides compression of the velocities in the case of linear stretching sheets and nonlinear stretching sheets. It is observed that the velocity for the linear stretching sheet is maximum and then as the stretching parameter increases the velocity decreases. Figure 6c,d revealed that the fluid velocity enhanced with the upshot of porosity parameter *K*, while reducing with the magnetic *M* effect. The variation in porosity term allows more particle to pass through the pores, which encourage fluid flow, on the other hand, the magnetic force produces an opposing and resistant effect to the fluid flow, which results in the retardation of the velocity field.

Figure 7a–d depicted the velocity profile behavior versus the nonlinear thermal convection $\sigma _{1}$, nonlinear mass convection $\sigma _{2}$, thermal Grashof number *Gr* and mass Grashof number *Gc* respectively. Figure 7a,b elaborated that the velocity field decrease with the rising effect of nonlinear thermal convection, while enhances with mass convection, because more reactant concentration is utilized by high exothermic reaction. Figure 7c,d revealed that the fluid velocity augmented with the effect of thermal and mass Grashof number. Physically, the stretching velocity of surface improves upon the variation of mass and thermal Grashof number, which results in the elevation of the velocity field.

Figure 8a–d display the comparative analysis of PCM and Matlab built in package bvp4c. It can be perceived that both the method are in best settlement with each other. For further accuracy and validity of the present numerical scheme, the outcomes of the PCM method are compared with already published work. Table 1 ensure the accuracy of the present numerical method upto 3 decimal places, which is a very good agreement with other numerical schemes.

Table 2 further exaggerate the validity of the present numerical results with already published work. The numerical values of the wall shear stress and heat transfer rate for both linear stretching sheet ($n=0$) and nonlinear stretching sheet ($n>0$) are tabulated in Table 2, which are very rational to the previously published work.

The computational cost of a numerical method play a very important role in the field of computational fluid dynamics, especially in nonlinear dynamics. The cost of computation depends on the cost per iteration and the number of iterations. The cost per iteration committed on the efficiency while the number of iterations depends on the accuracy of the method. Keeping in view this importance of the computational method, Table 3 illustrates the CPU time for both PCM and bvp4c for different values of the nonlinear parameter *n*. It can be observed that in each case the CPU time for PCM is shorter than bvp4c, so PCM is preferred over bvp4c.

**Figure 9:**
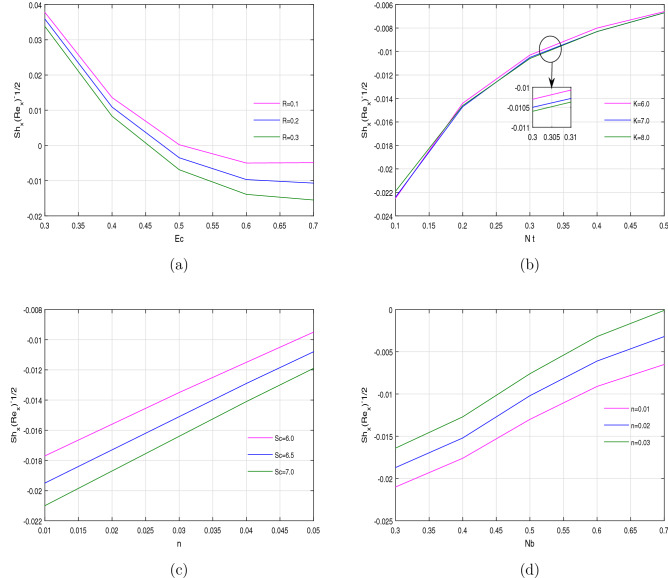
Variation of Sherwood number against different physical parameters.

**Figure 10:**
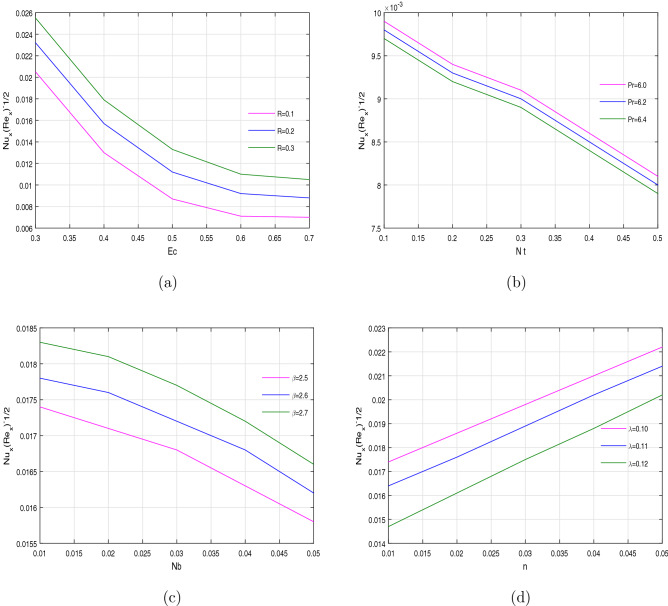
Variation of Nusselt number against different physical parameters.

**Figure 11:**
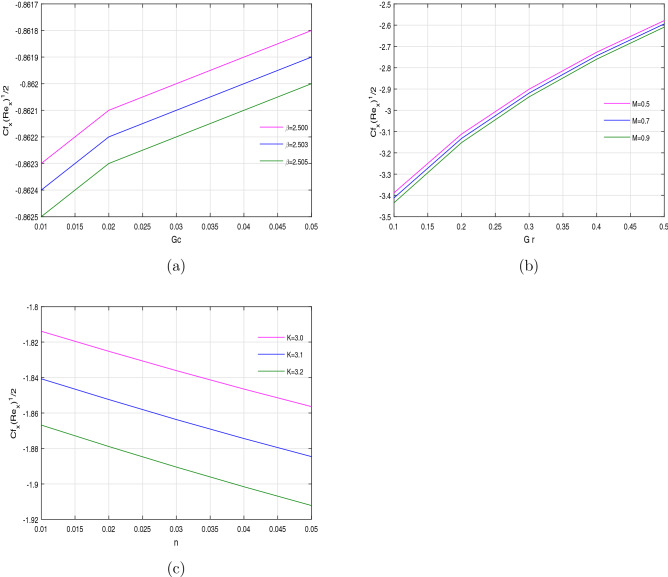
Variation of wall shear stress against different physical parameters.

The physical important quantities, such as mass transfer $Sh_{x}(Re_{x})^{-1/2}$, heat transfer $Nu_{x}(Re_{x})^{-1/2}$ rates and the rate of shear stress $Cf_{x}(Re_{x})^{1/2}$ have an important role in civil and mechanical engineering and even in soil mechanics and foundation engineering. Due to this wide range of applications of such quantities, the numerical results are elaborated graphically in Figs. 9, 10 and 11 against various parameters. Figure 9a–d shows a decreasing effect of the mass transfer rate for chemical reaction parameter *R*, porous medium parameter *k* and Schmidt number *Sc*, whilst an increasing in mass transfer rate for power law index *n*. It can be reason out that the internal energy of the fluid particles is increased by the chemical reaction parameter *R*, which enhance the heat transfer rate and as a result consume more concentration and decline the mass transfer rate. Schmidt number *Sc* has a direct relation with fluid’s viscosity $\nu$, which cause an increase in mass transfer rate.

The heat transfer rate $Nu_{x}(Re_{x})^{-1/2}$ can be depicted in Fig. 10a–d. The heat transfer rate is increased for both the chemical reaction parameter *R* Fig. 10a, and Casson fluid parameter $\beta$ Fig. 10c, against Eckert number *Ec* and Brownian motion parameter *Nb*, respectively. One can see that the Casson parameter has an inverse relation with fluid’s viscosity within temperature equation. Due to this inverse relation, the dynamic viscosity will be decreased with the increasing values of $\beta$ and as a result much more heat will be transferred. Figure 10b,d shows that the heat transfer rate decline for the increasing values of Prandtl number *Pr* and heat source parameter $\lambda$, respectively. This increase in heat transfer rate is due to the reduction of thermo diffusivity of the fluid by Prandtl number.

The wall shear stress is the uniform distribution of fluid’s flux throughout the surface. This uniform flux is a result of electromagnetic induction which carries a uniform time dependent current. The Casson parameter $\beta$ behaves like a non-Newtonian liquid, upto certain extent, which creates a resistant to fluid’s flux, due to fluid’s viscosity. That is the reason for the reduction of the wall shear stress for the increasing values of $\beta$ versus concentration buoyancy *Gc* (Fig. 11a). Magnetic filed is usually used to control the turbulence behavior of the fluid in fluid mechanics. Magnetic field create an opposing like force, called Lorentz force, against the fluid flow motion. This is the reason that the increasing values of the magnetic parameter *M* increases the frictional force on the surface, which results a reduction of the wall shear stress (Fig. 11b). The variation of the wall shear stress against porosity parameter *k* and power law index *n* is plotted in Fig. 11c. The increasing values of the porosity parameter intercept the fluid’s velocity, which causes a reduction in the wall shear stress.

**Table 1:** Comparison of the present result with previous published work, when $Ec=f_{w}=\lambda =0$ and for present work $\varphi =K = R =Gr=Gc= 0$.

Constants						Reference^41^	Reference^6^	Present work
n	Nt	Pr	Sc	$\beta$	M	$-\theta ' (0)$	$-\theta ' (0)$	$-\theta ' (0)$
0.5	0.1	7	20	0.6	3	2.12068	2.12064	2.12066
0.5	0.5	7	20	0.6	3	1.63864	1.63865	1.63867
0.5	0.9	7	20	0.6	3	1.37927	1.37929	1.37925
1	0.5	7	5	0.6	3	2.69156	2.69158	2.69155
1	0.5	7	10	0.6	3	2.51354	2.51355	2.51357
1	0.5	7	20	0.6	3	2.31554	2.31559	2.31557
1.5	0.5	5	20	0.6	3	2.43078	2.43075	2.43073
1.5	0.5	7	20	0.6	3	2.85270	2.85266	2.85268
1.5	0.5	9	20	0.6	3	3.21459	3.21451	3.21455
2	0.5	7	20	0.2	3	3.41143	3.41132	3.41140
2	0.5	7	20	0.6	3	3.31040	3.31030	3.31041
2	0.5	7	20	1.0	3	3.26319	3.26311	3.26315
3	0.5	7	20	0.6	0	4.18830	4.18829	4.18833
3	0.5	7	20	0.6	1	4.14820	4.14822	4.14818
3	0.5	7	20	0.6	3	4.08219	4.08222	4.08224

**Table 2:** Comparison of the present result with previous published work, when $Ec=f_{w}=\lambda =0$ and for present work $\varphi = K = R =Gr=Gc= 0$.

n	Reference^42^		Reference^43^		Reference^44^		Present work	
	$-f''(0)$	$-\theta ' (0)$	$-f''(0)$	$-\theta ' (0)$	$-f''(0)$	$-\theta ' (0)$	$-f''(0)$	$-\theta ' (0)$
0	0.627547	–	0.6276	–	0.627556	–	0.6265	–
0.2	0.766758	0.610262	0.7668	0.6102	0.766838	0.610203	0.7672	0.61032
0.5	0.889477	0.595277	0.8896	0.5949	0.889545	0.595204	0.8883	0.5960
1	1.0	–	1.0	–	1.000001	–	1.0000	–
3	1.148588	0.564472	1.1486	0.5647	1.148594	0.564670	1.14867	0.5637
10	1.234875	0.554960	1.2349	0.5549	1.234876	0.554890	1.2336	0.5537
100	1.276768	–	1.2768	–	1.276775	–	1.2755	–

**Table 3:** CPU time for two different techniques.

n	CPU time for PCM (s)	CPU time for bvp4c (s)
1	1.306	2.078
2	1.322	1.699
3	1.302	1.751
4	1.469	1.985
5	1.357	1.974
6	1.098	1.881

## Conclusion

The computational assessment has been made for the viscoelastic Casson fluid over a permeable stretchable surface. Exothermic chemical reactions, heat generation effect, magnetic field and nonlinear volumetric thermal/mass expansion are the major contribution of the current analysis. The proposed system of PDEs, which are responsible for fluid motion, are transformed to the dimensionless system of ODEs by using a suitable transformation. The obtained set of differential equations are numerically computed through PCM procedure. The succeeding outcomes are the key points.

- The velocity profile is increases with mass convection $\sigma _{2}$, because more reactant concentration is utilized by high exothermic reaction.
- Physically, the stretching velocity of surface improves upon the variation of mass and thermal Grashof numbers *Gr* and *Gc*, respectively, which results in the elevation of the velocity field.
- The internal energy of the fluid particles is increased by the chemical reaction parameter *R*, which enhance the heat transfer rate and as a result consume more concentration and decline the mass transfer rate.
- Schmidt number *Sc* has a direct relation with fluid’s viscosity $\nu$, which cause an increase in mass transfer rate.
- Due to the inverse relation, the dynamic viscosity will be decreased with the increasing values of $\beta$ and as a result much more heat will be transferred.
- The increasing values of the porosity parameter intercept the fluid’s velocity, which causes a reduction in the wall shear stress.

## Data Availability

The datasets used and analyzed during the current study available from the corresponding author on reasonable request.

## List of symbols

$\beta$: Casson fluid constraint
$$\lambda$$: Heat source
$$\phi$$: Dimensionless concentration
$$\varphi$$: Inclined angle
$B_{0}$: Magnetic induction (tesla (T))
$C_{f}$: Skin-friction coefficient
*Ec*: Eckert number
*f*: Dimensionless stream function
*Gc*: Buoyancy concentration (N)
*Gr*: Thermal buoyancy (N)
*K*: Porosity term
*k*: Surface permeability
*M*: Magnetic effect (tesla T)
$$N_{b}$$: Brownian motion
$$N_{t}$$: Thermophoresis
$Nu_{x}$: Local Nusselt number
*Pr*: Prandtl number
$$Q_{0}$$: Absorption coefficient
$R_{0}$: Chemical reaction term
$Re_{x}$: Local Reynolds number
$Sh_{x}$: Local Sherwood number
$$T_{\infty }$$: Ambient temperature
$$T_{w}$$: Reference temperature
$$u_{w}$$: Reference velocity
$\infty$: Conditions at infinity
$$\omega$$: Surface conditions
$\tau$: Heat capacity ratio (JK$^{-1}$)
*C*: Mass concentration (g L$$^{-1}$$)
$$C_{\infty }$$: Ambient concentration (g L$$^{-1}$$)
$C_{p}$: Specific heat capacity (J Kg K$^{-1}$)
*n*: Power law index
*Sc*: Schmidt number
